# Supplementary material for: Deletion of wfs1 Impairs Oligodendrocyte Precursor Cells Dorsal Distribution and Myelination Through the wfs1-hmgcs1 Axis in Zebrafish
Source: Biology (Basel). 2026 Aug 16;15(16):1402. doi: 10.3390/biology15161402 (PMC13510151; doi:10.3390/biology15161402)
Supplement: Supplementary file 1 [file biology-15-01402-s001.zip › File S1-Transcriptome sequencing data and differentially expressed genes (DEGs) in wfs1-- zebrafish at 5 dpf/2.KEGG/wfs1KOvsWT/GSEA/WT/SPHINGOLIPID_METABOLISM/SPHINGOLIPID_METABOLISM(DRE00600).html]

Details for gene set SPHINGOLIPID\_METABOLISM(DRE00600)[GSEA]

|  || Dataset | deg.sample |
| Phenotype | sample.cls |
| Upregulated in class | WT |
| GeneSet | SPHINGOLIPID\_METABOLISM(DRE00600) |
| Enrichment Score (ES) | -0.2617516 |
| Normalized Enrichment Score (NES) | -1.2187823 |
| Nominal p-value | 0.09756097 |
| FDR q-value | 0.4655522 |
| FWER p-Value | 1.0 |
Table: GSEA Results Summary

  

Fig 1: Enrichment plot: SPHINGOLIPID\_METABOLISM(DRE00600)      
 Profile of the Running ES Score & Positions of GeneSet Members on the Rank Ordered List

  

| PROBE | DESCRIPTION (from dataset) | GENE SYMBOL | GENE\_TITLE | RANK IN GENE LIST | RANK METRIC SCORE | RUNNING ES | CORE ENRICHMENT || 1 | 450083 | gla |  |  | 814 | 2.368 | 0.0223 | No |
| 2 | 550266 | acer1 |  |  | 1565 | 1.614 | 0.0314 | No |
| 3 | 493602 | asah2 |  |  | 1865 | 1.427 | 0.0509 | No |
| 4 | 793635\_1 | b4galnt1b |  |  | 2414 | 1.203 | 0.0580 | No |
| 5 | 553981 | sptlc1 |  |  | 2420 | 1.200 | 0.0822 | No |
| 6 | 559124 | ppap2d |  |  | 2971 | 1.043 | 0.0860 | No |
| 7 | 724080 | b4galt5 |  |  | 3003 | 1.035 | 0.1059 | No |
| 8 | 556439 | si:ch211-212g7.6 |  |  | 3230 | 0.987 | 0.1188 | No |
| 9 | 559072 | gba |  |  | 3533 | 0.923 | 0.1280 | No |
| 10 | 394114 | kdsr |  |  | 4175 | 0.797 | 0.1240 | No |
| 11 | 492359\_1 | zgc:91976 |  |  | 4674 | 0.723 | 0.1230 | No |
| 12 | 797914 | smpd2b |  |  | 4771 | 0.709 | 0.1343 | No |
| 13 | 100037312 | sgpl1 |  |  | 5110 | 0.666 | 0.1371 | No |
| 14 | 559124\_1 | ppap2d |  |  | 5172 | 0.659 | 0.1486 | No |
| 15 | 563806 | plpp2a |  |  | 6278 | 0.559 | 0.1251 | No |
| 16 | 553508\_1 | ugt8 |  |  | 6332 | 0.554 | 0.1347 | No |
| 17 | 558684 | b4galnt1a |  |  | 7354 | 0.463 | 0.1119 | No |
| 18 | 548343 | glb1 |  |  | 7702 | 0.440 | 0.1099 | No |
| 19 | 402799 | degs2 |  |  | 7971 | 0.425 | 0.1101 | No |
| 20 | 335485 | plpp2b |  |  | 8056 | 0.419 | 0.1159 | No |
| 21 | 557817\_2 | enpp7.2 |  |  | 8197 | 0.415 | 0.1199 | No |
| 22 | 557756 | enpp7.1 |  |  | 8256 | 0.414 | 0.1265 | No |
| 23 | 568169 | cers1 |  |  | 8910 | 0.374 | 0.1135 | No |
| 24 | 568381\_1 | smpd3 |  |  | 9129 | 0.358 | 0.1139 | No |
| 25 | 557680 | plpp3 |  |  | 9279 | 0.348 | 0.1163 | No |
| 26 | 557756\_2 | enpp7.1 |  |  | 9331 | 0.345 | 0.1217 | No |
| 27 | 393549 | asah1b |  |  | 9770 | 0.317 | 0.1143 | No |
| 28 | 568381 | smpd3 |  |  | 9809 | 0.315 | 0.1195 | No |
| 29 | 793735 | gal3st1a |  |  | 9966 | 0.305 | 0.1208 | No |
| 30 | 569930\_1 | zgc:153896 |  |  | 10686 | 0.261 | 0.1034 | No |
| 31 | 793584 | gal3st1b |  |  | 10746 | 0.258 | 0.1068 | No |
| 32 | 323020 | cers5 |  |  | 10762 | 0.257 | 0.1115 | No |
| 33 | 550460 | hexa |  |  | 11206 | 0.233 | 0.1023 | No |
| 34 | 259250 | cers4a |  |  | 11402 | 0.222 | 0.1007 | No |
| 35 | 393912 | b4galt6 |  |  | 12226 | 0.178 | 0.0784 | No |
| 36 | 323613 | hexb |  |  | 12362 | 0.171 | 0.0776 | No |
| 37 | 567592 | smpd5 |  |  | 12443 | 0.167 | 0.0785 | No |
| 38 | 449649 | galca |  |  | 12897 | 0.146 | 0.0672 | No |
| 39 | 553646 | sptlc2a |  |  | 13078 | 0.136 | 0.0643 | No |
| 40 | 793635 | b4galnt1b |  |  | 13834 | 0.100 | 0.0426 | No |
| 41 | 259251 | cers2a |  |  | 14222 | 0.081 | 0.0320 | No |
| 42 | 445168 | sptlc3 |  |  | 14458 | 0.071 | 0.0261 | No |
| 43 | 557286 | sptlc2b |  |  | 14508 | 0.069 | 0.0260 | No |
| 44 | 100332380 | cers3a |  |  | 14629 | 0.064 | 0.0235 | No |
| 45 | 100000684 | neu3.5 |  |  | 14952 | 0.049 | 0.0143 | No |
| 46 | 100331840 | sphk1 |  |  | 15103 | 0.041 | 0.0104 | No |
| 47 | 794598 | LOC794598 |  |  | 15191 | 0.038 | 0.0085 | No |
| 48 | 566563 | sgms1 |  |  | 15313 | 0.032 | 0.0053 | No |
| 49 | 100330698 | sgpp2 |  |  | 15386 | 0.028 | 0.0036 | No |
| 50 | 556193 | sgms2 |  |  | 16241 | -0.009 | -0.0231 | No |
| 51 | 450068 | asah1a |  |  | 16546 | -0.019 | -0.0322 | No |
| 52 | 559240 | gba2 |  |  | 16594 | -0.020 | -0.0333 | No |
| 53 | 327075 | degs1 |  |  | 16838 | -0.028 | -0.0404 | No |
| 54 | 140811 | psap |  |  | 17418 | -0.050 | -0.0576 | No |
| 55 | 558496 | cerk |  |  | 17489 | -0.053 | -0.0587 | No |
| 56 | 567548 | smpd2a |  |  | 17857 | -0.070 | -0.0688 | No |
| 57 | 557817\_1 | enpp7.2 |  |  | 18126 | -0.082 | -0.0756 | No |
| 58 | 100003745 | sgpp1 |  |  | 18308 | -0.089 | -0.0795 | No |
| 59 | 556700 | smpd1 |  |  | 18519 | -0.100 | -0.0841 | No |
| 60 | 101884228 | LOC101884228 |  |  | 18589 | -0.103 | -0.0841 | No |
| 61 | 569930 | zgc:153896 |  |  | 18818 | -0.114 | -0.0890 | No |
| 62 | 567592\_1 | smpd5 |  |  | 19076 | -0.125 | -0.0946 | No |
| 63 | 553508 | ugt8 |  |  | 19174 | -0.130 | -0.0950 | No |
| 64 | 406385 | galcb |  |  | 19487 | -0.143 | -0.1019 | No |
| 65 | 541416 | arsa |  |  | 19811 | -0.158 | -0.1088 | No |
| 66 | 557756\_1 | enpp7.1 |  |  | 20424 | -0.184 | -0.1244 | No |
| 67 | 100141367 | cers3b |  |  | 20602 | -0.194 | -0.1260 | No |
| 68 | 569053 | plpp1a |  |  | 21219 | -0.226 | -0.1408 | No |
| 69 | 100150090 | sphk2 |  |  | 22316 | -0.285 | -0.1695 | No |
| 70 | 492359 | zgc:91976 |  |  | 22557 | -0.299 | -0.1710 | No |
| 71 | 450083\_1 | gla |  |  | 23329 | -0.350 | -0.1882 | No |
| 72 | 555206\_1 | neu3.3 |  |  | 23654 | -0.371 | -0.1908 | No |
| 73 | 559850 | neu1 |  |  | 23773 | -0.378 | -0.1869 | No |
| 74 | 565295 | cers2b |  |  | 24254 | -0.411 | -0.1937 | No |
| 75 | 564867 | cers6 |  |  | 25216 | -0.461 | -0.2146 | No |
| 76 | 553569 | neu4 |  |  | 25521 | -0.486 | -0.2143 | No |
| 77 | 793294 | smpd4 |  |  | 25959 | -0.526 | -0.2174 | No |
| 78 | 553944 | ugcg |  |  | 26190 | -0.546 | -0.2136 | No |
| 79 | 793046\_1 | neu3.4 |  |  | 26386 | -0.566 | -0.2082 | No |
| 80 | 791197 | zgc:158263 |  |  | 26866 | -0.615 | -0.2109 | No |
| 81 | 780844 | neu3.1 |  |  | 27740 | -0.708 | -0.2240 | No |
| 82 | 555411 | acer3 |  |  | 28300 | -0.786 | -0.2257 | No |
| 83 | 100331179 | acer2 |  |  | 29449 | -1.046 | -0.2406 | Yes |
| 84 | 335485\_1 | plpp2b |  |  | 29460 | -1.049 | -0.2197 | Yes |
| 85 | 553981\_1 | sptlc1 |  |  | 30460 | -1.483 | -0.2211 | Yes |
| 86 | 100330155 | cers4b |  |  | 30886 | -1.848 | -0.1970 | Yes |
| 87 | 100000684\_1 | neu3.5 |  |  | 30984 | -1.951 | -0.1606 | Yes |
| 88 | 445250 | neu3.2 |  |  | 31793 | -4.548 | -0.0939 | Yes |
| 89 | 445250\_1 | neu3.2 |  |  | 31824 | -4.790 | 0.0021 | Yes |
Table: GSEA details [plain text format]

  

Fig 2: SPHINGOLIPID\_METABOLISM(DRE00600)      
 Blue-Pink O' Gram in the Space of the Analyzed GeneSet

  

Fig 3: SPHINGOLIPID\_METABOLISM(DRE00600): Random ES distribution      
 Gene set null distribution of ES for **SPHINGOLIPID\_METABOLISM(DRE00600)**
